# Supplementary material for: The NAC transcription factors SNAP1/2/3/4 are central regulators mediating high nitrogen responses in mature nodules of soybean
Source: Nat Commun. 2023 Aug 5;14:4711. doi: 10.1038/s41467-023-40392-w (PMC10404276; doi:10.1038/s41467-023-40392-w)
Supplement: Supplementary file 1 — Supplementary Information [file 41467_2023_40392_MOESM1_ESM.pdf]

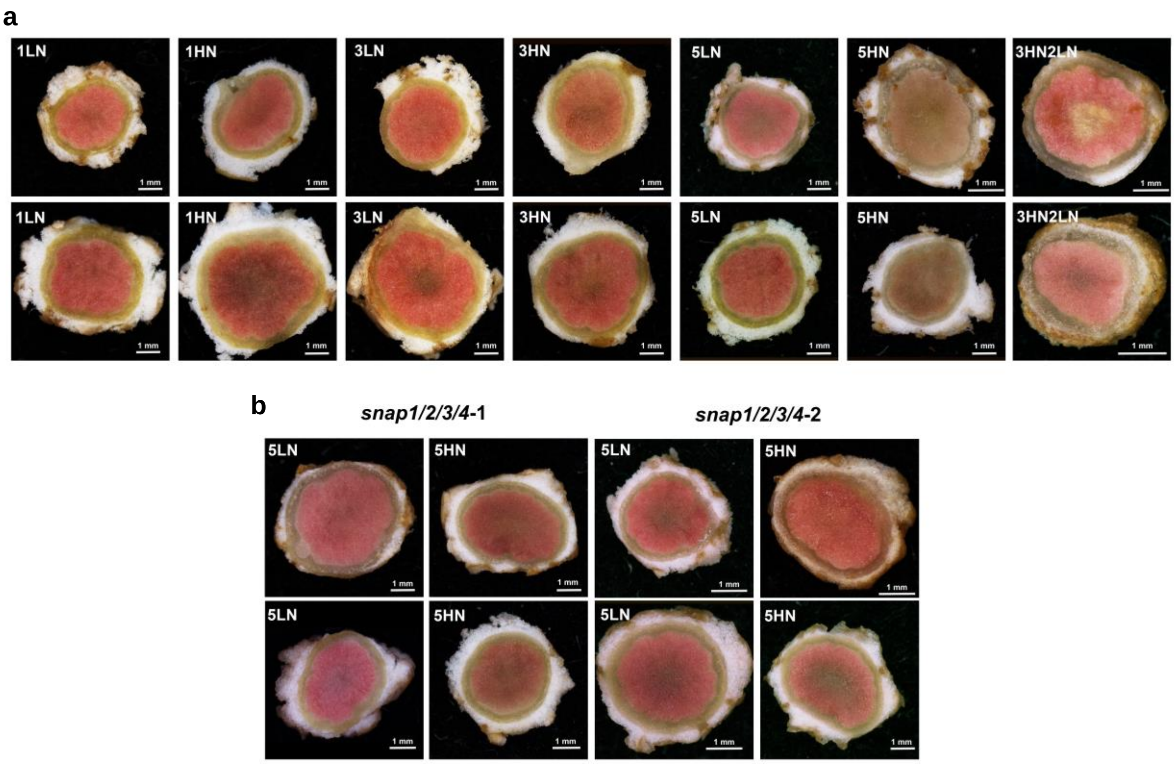


**Supplementary Figure 1.** Cross section observations of mature nodules from **a** WT and **b** two *snap1/2/3/4* mutants under different N treatments. Scale bars = 1 mm. For each sample, two sections were shown. HN: high N treatment; LN: low N treatment.

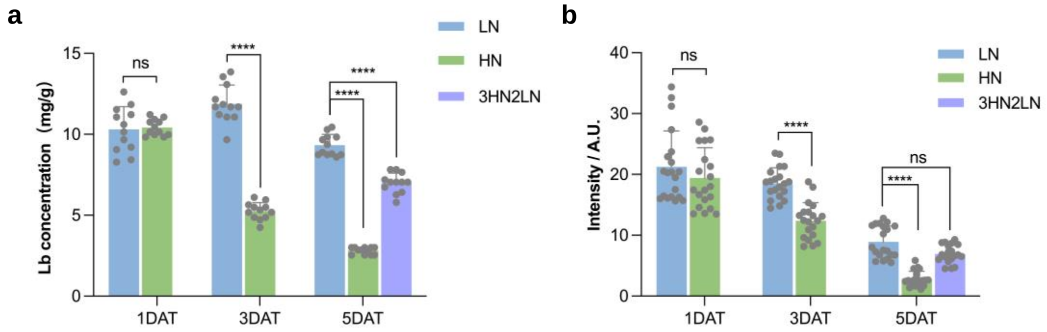


**Supplementary Figure 2.** Statistical summary of **a** Leghemoglobin content and **b** fluorescence intensity of nodule samples under different N treatments. All data are represented as mean ± SD. n=12 and n=20 are calculated for Leghemoglobin content and fluorescence intensity, respectively. DAT: day after treatment. HN: high N treatment; LN: low N treatment; ns: not significant. Statistically significant differences were performed using Student's t-test (two-tailed). ****P < 0.0001. Source data are provided as a Source Data file.

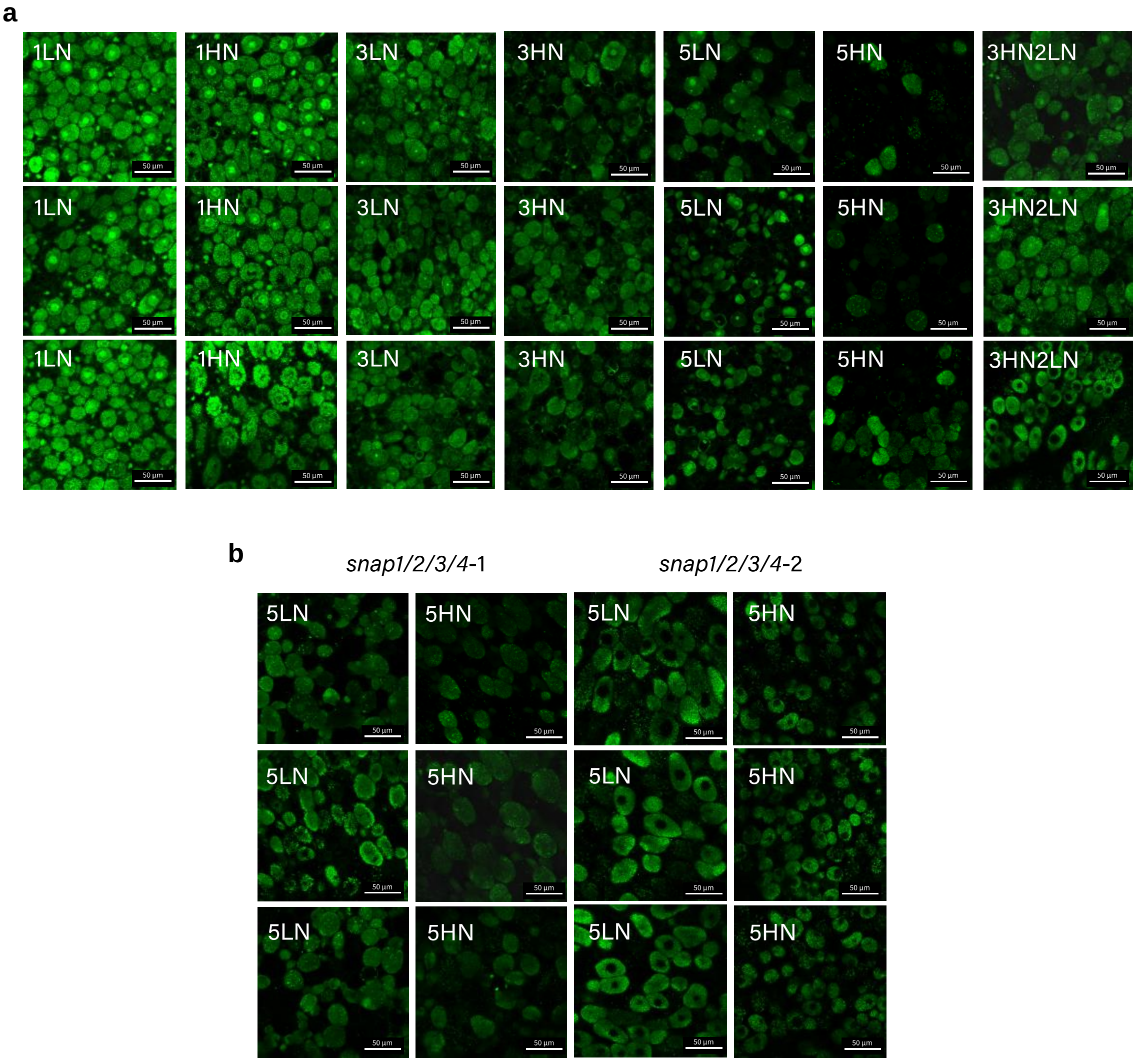


**Supplementary Figure 3.** SYTO9 staining for rhizobia in mature nodules of **a** WT and **b** two *snap1/2/3/4* mutants under different N treatments. Scale bars = 50 μm. For each sample, three sections were shown. HN: high N treatment; LN: low N treatment. Two replicates were performed for each experiment.

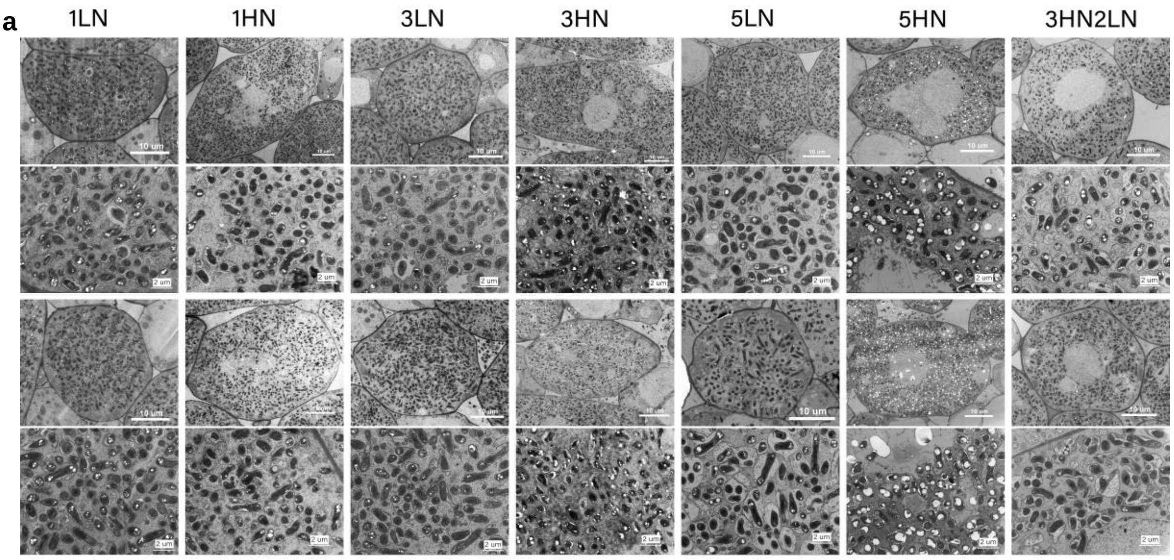


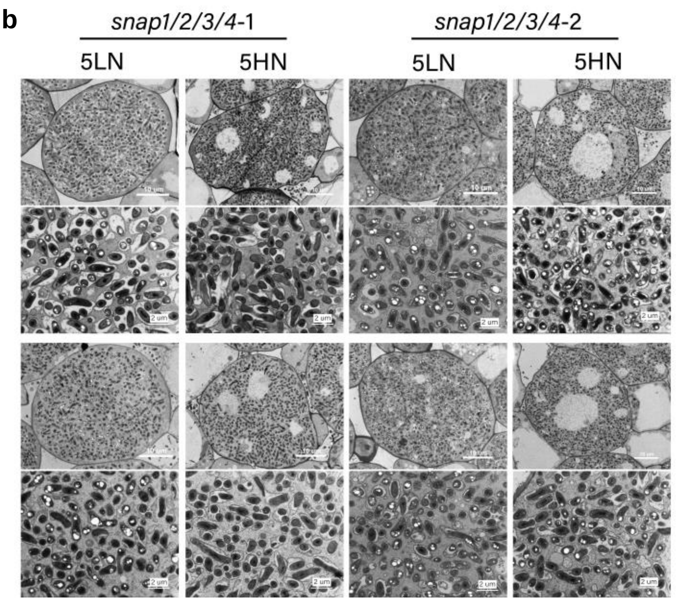


**Supplementary Figure 4.** Transmission electron micrographs of mature nodules of **a** WT and **b** two *snap1/2/3/4* mutants under different N treatments. For each sample, two micrographs were shown with two magnifications. Up panel: Scale bar = 10 μm; down panel: Scale bar = 2 μm. HN: high N treatment; LN: low N treatment. Two replicates were performed for each experiment.

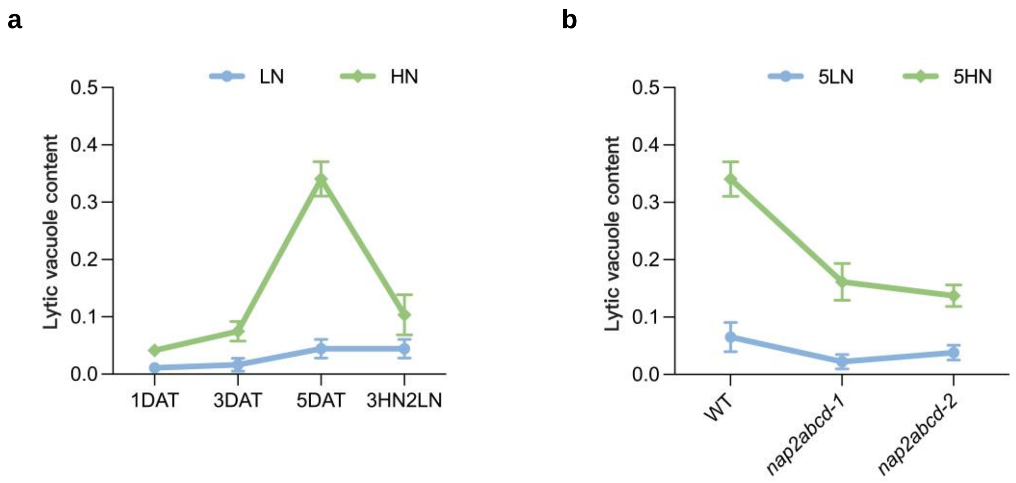


**Supplementary Figure 5.** Relative lytic vacuole content of **a** WT and **b** two *snap1/2/3/4* mutants under different N treatments. All data are represented as mean ± SEM (n=10). DAT: day after treatment. HN: high N treatment; LN: low N treatment. The relative vacuole content was calculated by the area of vacuole/the area of cell. Source data are provided as a Source Data file.

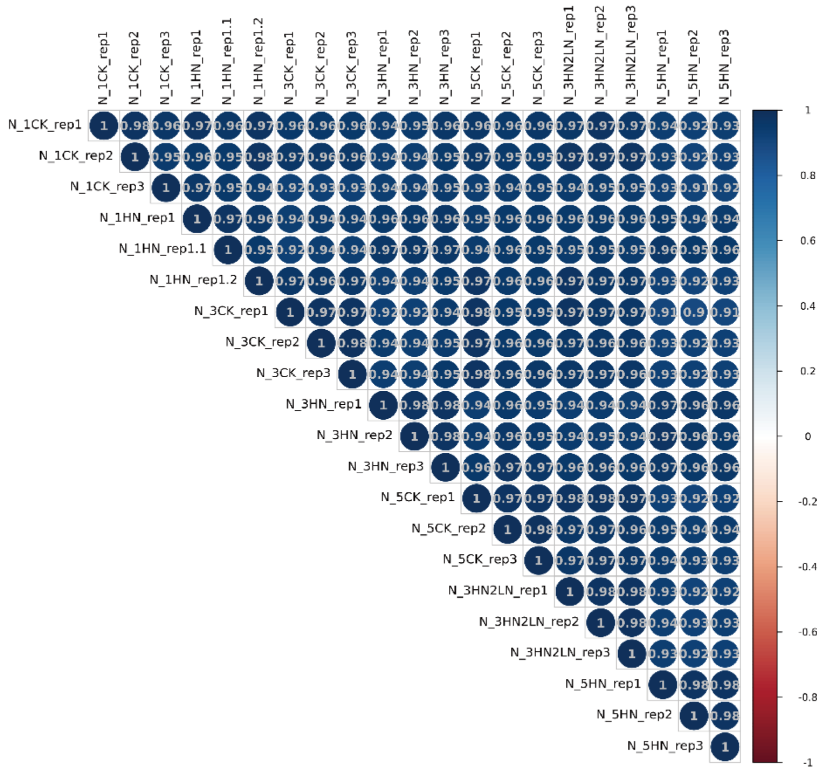


**Supplementary Figure 6.** Pairwise Spearman correlation coefficient analysis of RNA-seq samples in time-series high N treatment. N: nodule, rep: replicate. Values represent Spearman correlation coefficient of all the genes tested for differential expression between low N and high N nodule RNA-seq samples.

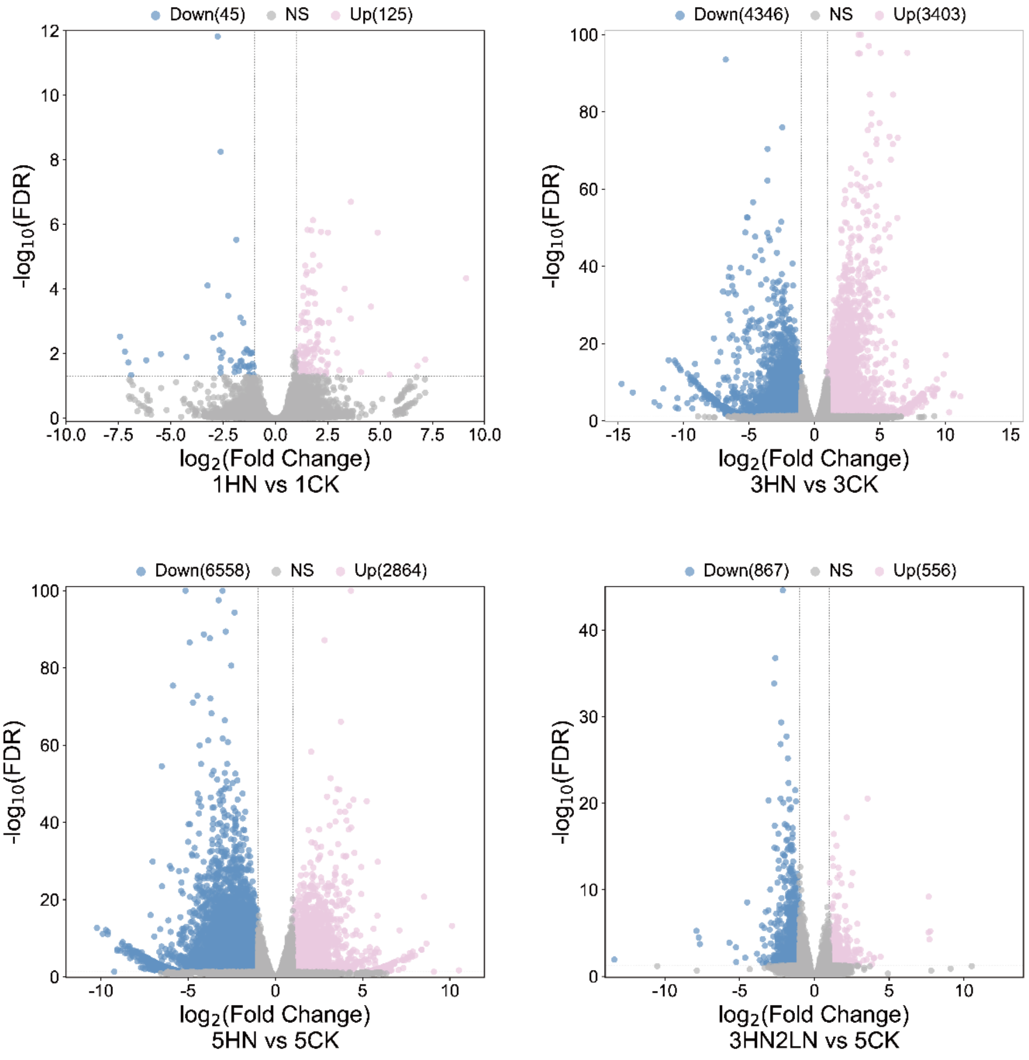


**Supplementary Figure 7.** Volcano plots of all expressed genes in time-series high N treatment. Dotted lines indicate $\log_2$(fold change) = 2 and FDR = 0.05 respectively. Down: down-expressed genes; Up: up-expressed genes. NS: not significant. Source data are provided as a Source Data file.

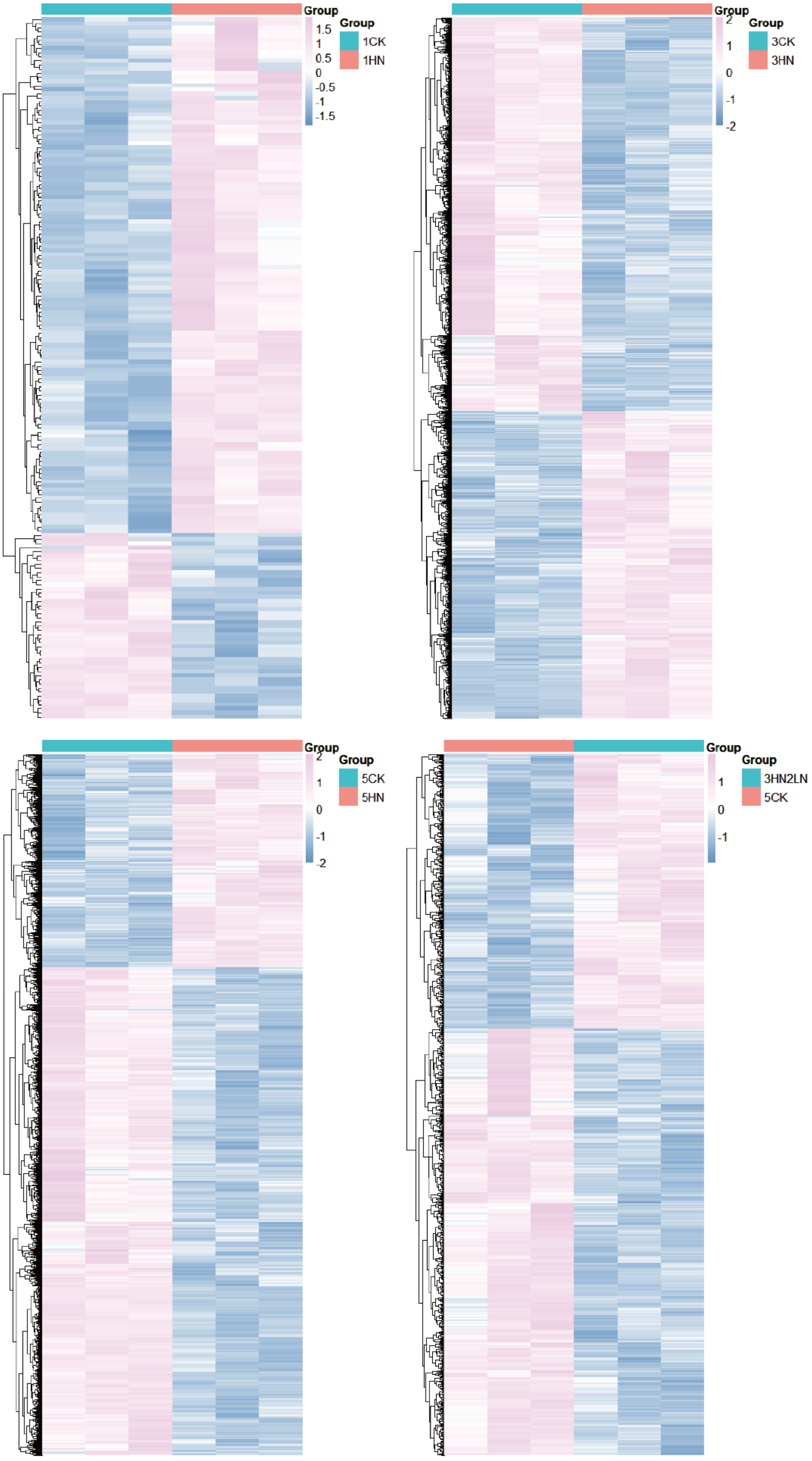


**Supplementary Figure 8.** Heatmaps of DEGs in time-series high N treatment. The scale is normalized with row values. Three biological replicates were shown. DEGs are determined by $log_2$(fold change) > 1 and FDR < 0.05.

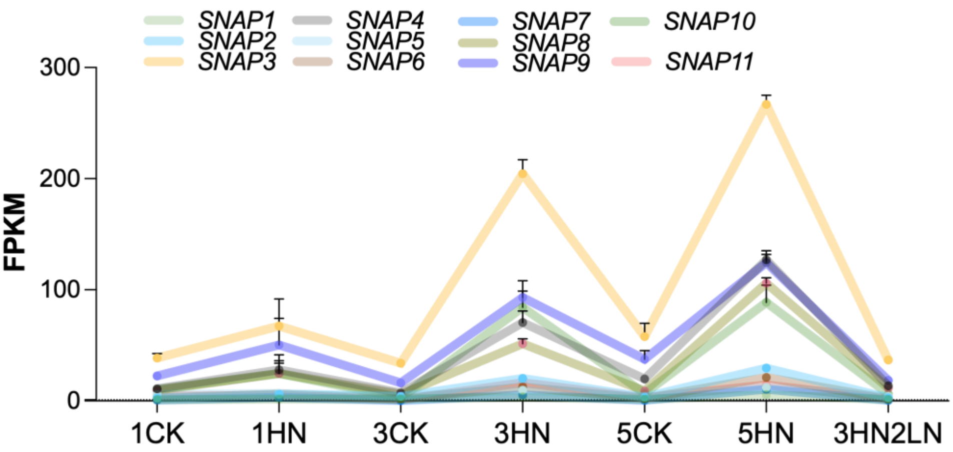


**Supplementary Figure 9.** Normalized expression level of soybean *SNAP* genes. Error bar: sem. FPKM: Fragments Per Kilobase of exon model per Million mapped fragments. Data are represented as average FPKM ± SEM of three biological replicates. Source data are provided as a Source Data file.

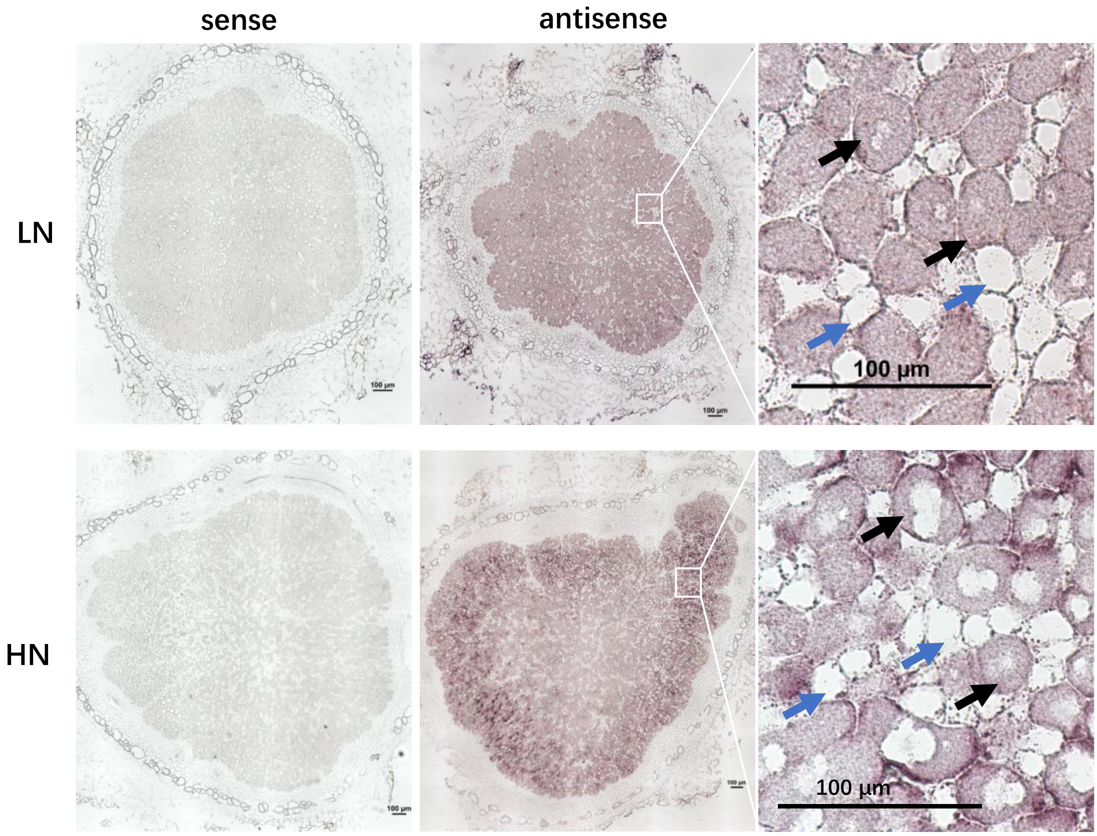


**Supplementary Figure 10.** In situ hybridization of *SNAP3* gene. Upper panel shows that *SNAP3* expressed in nodule under LN condition and lower panel shows that *SNAP3* expressed in nodule under 5HN treatment. Black arrows indicate infected cell and blue arrows indicate uninfected cell.

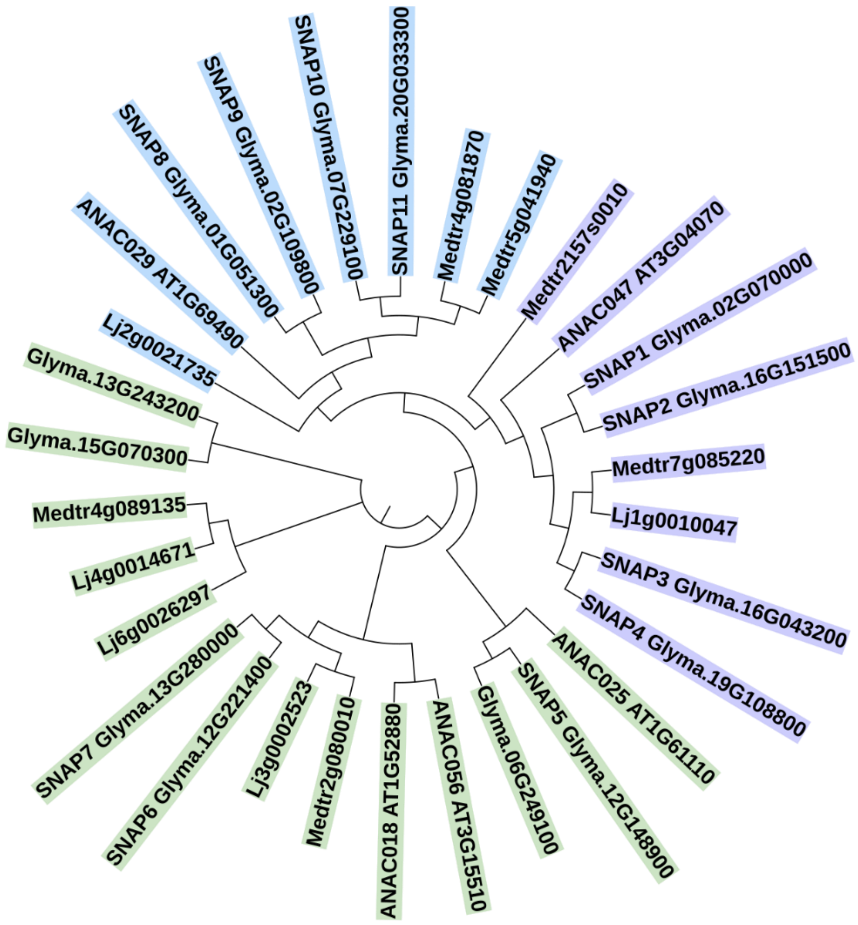


**Supplementary Figure 11.** Phylogenetic tree of 11 soybean SNAP TFs and their homologs in *Medicago truncatula*, *lotus japonicus* and Arabidopsis. Genes in the same colors indicate they are in the same clade.

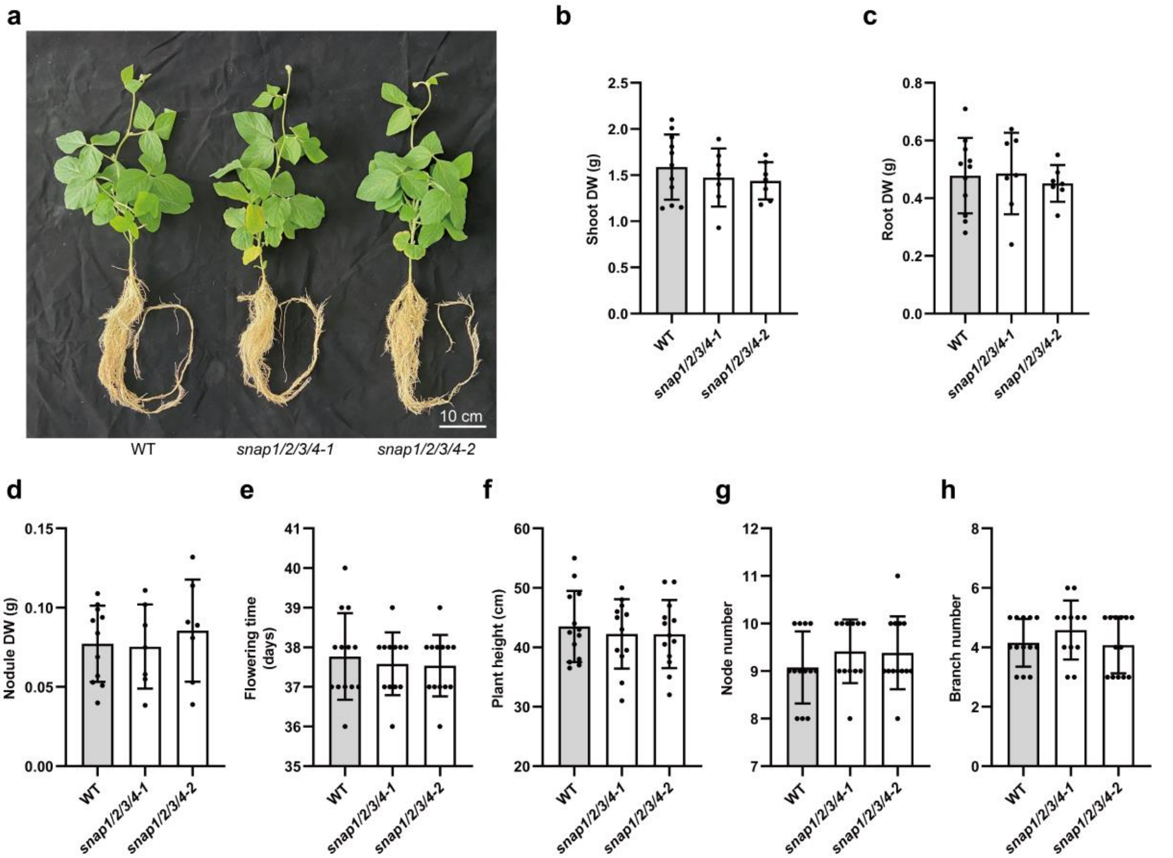


**Supplementary Figure 12.** Phenotypes of two *snap1/2/3/4* mutants and WT under LN condition. **a** Two *snap1/2/3/4* mutants and WT seedlings cultivated in hydroponics under LN condition. Photos were taken at 43 DAG (days after germination). Phenotypic summary of **b** shoot DW, **c** root DW, **d** nodule DW, **e** flowering time, **f** plant height, **g** node number and **h** branch number. DW: dry weight. All data are represented as mean ± SD. For shoot DW, root DW and nodule DW, at least 7 plants were calculated for WT, *snap1/2/3/4-1* and *snap1/2/3/4-2* at 35 DAG. For flowering time, plant height, node number and branch number, at least 12 plants were calculated for WT, *snap1/2/3/4-1* and *snap1/2/3/4-2* at 43 DAG. Source data are provided as a Source Data file.

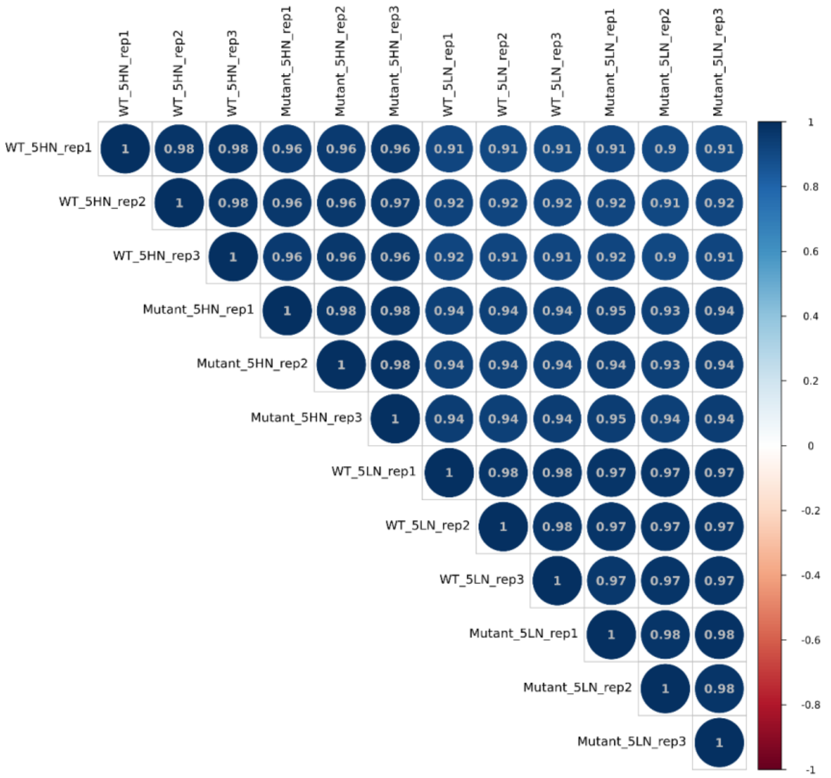


**Supplementary Figure 13.** Pairwise Spearman correlation coefficient analysis of RNA-seq samples of WT and *snap1/2/3/4-1* mutant under 5HN and 5LN condition. rep: replicate. Mutant: *snap1/2/3/4-1*. Values represent Spearman correlation coefficient of all the genes tested for differential expression between low N and high N nodule RNA-seq samples.

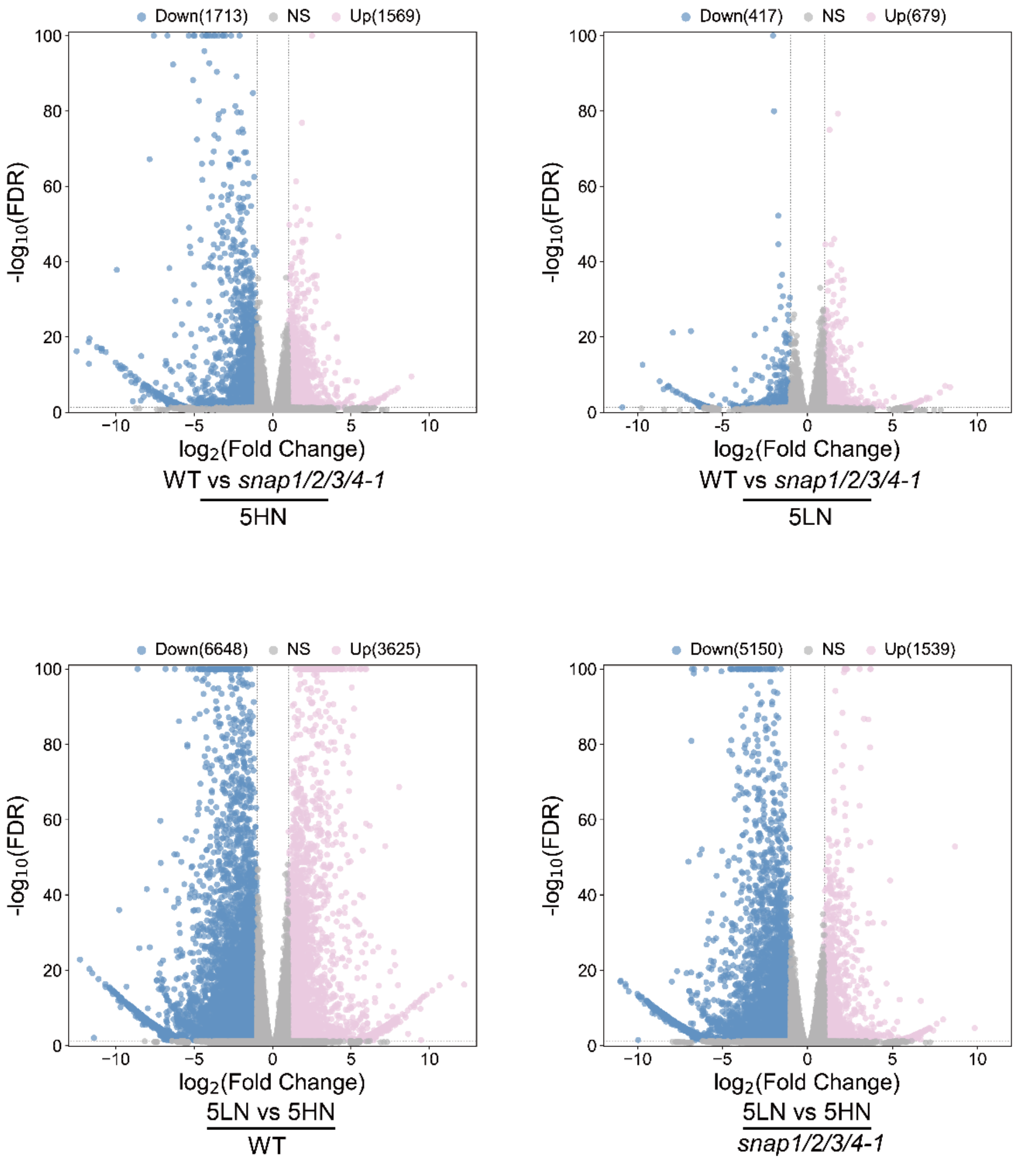


**Supplementary Figure 14.** Volcano plot of all expressed genes in WT and *snap1/2/3/4-1* mutant under 5HN and 5LN condition. Dotted lines indicate $\log_2$(fold change) = 2 and FDR = 0.05 respectively. Down: down-expressed genes; Up: up-expressed genes. NS: not significant. Source data are provided as a Source Data file.

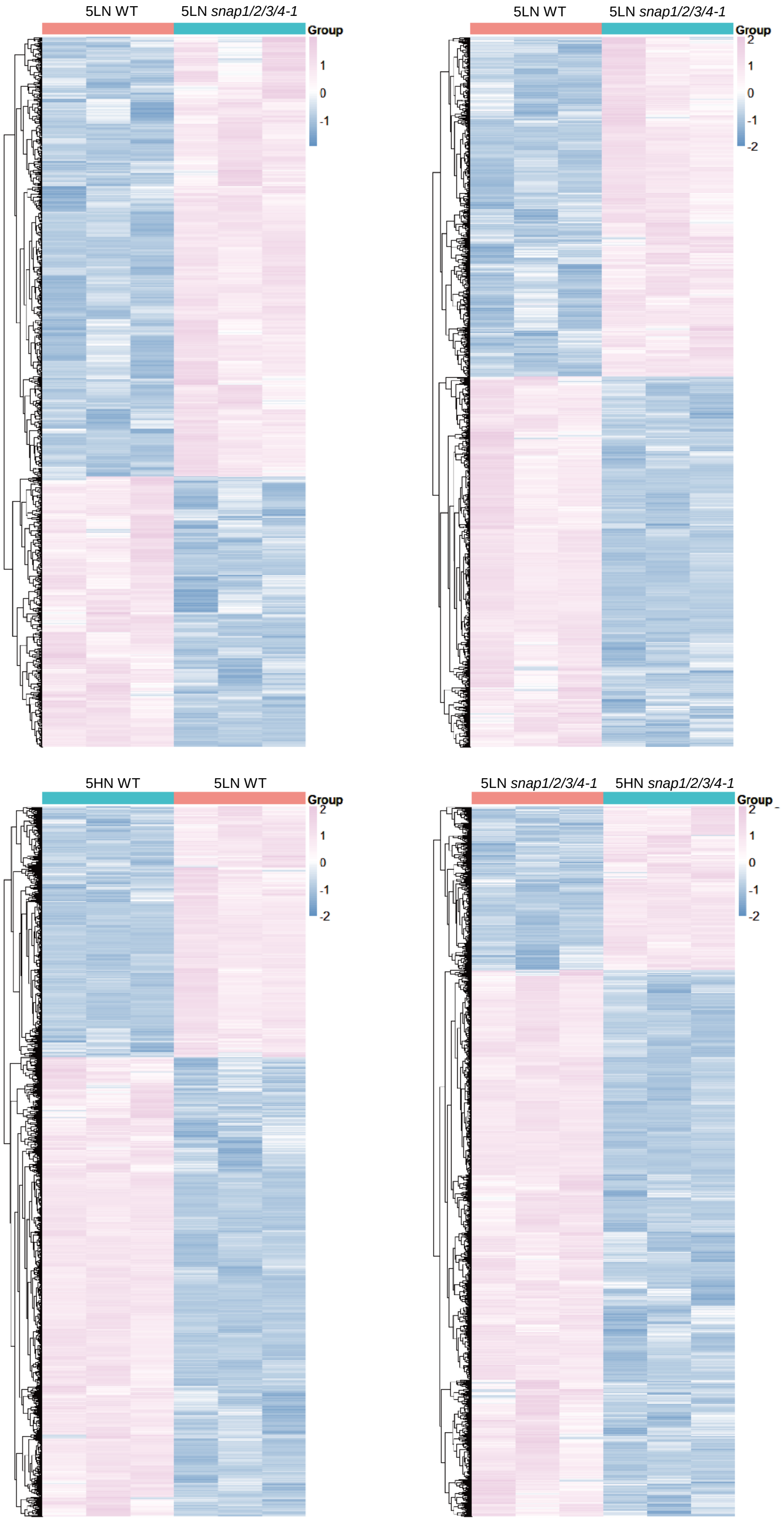


**Supplementary Figure 15.** Heatmaps of DEGs WT and *snap1/2/3/4-1* mutant under 5HN and 5LN condition. The scale is normalized with row values. Three biological replicates are shown. DEGs are determined by $\log_2$(fold change) > 1 and FDR < 0.05.

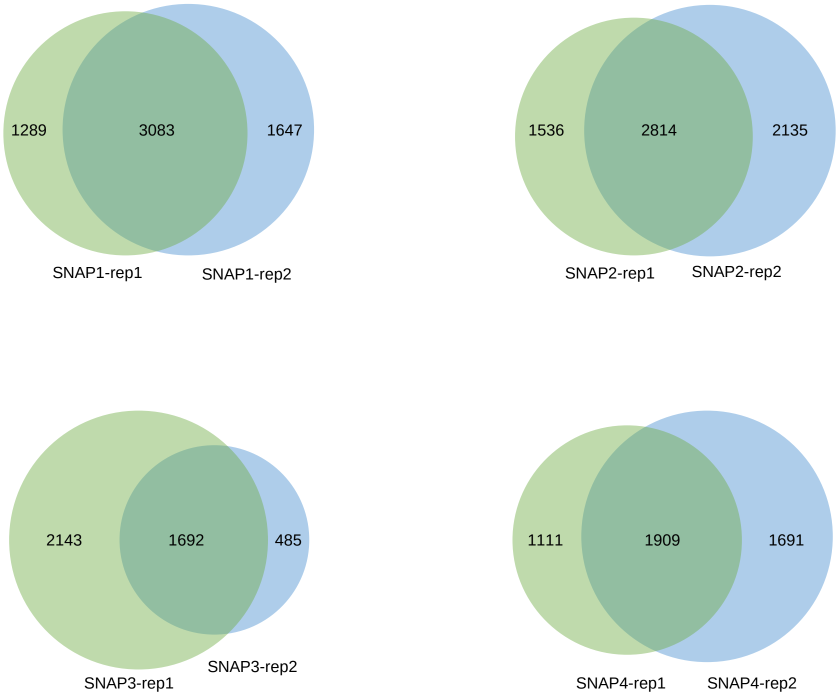


**Supplementary Figure 16.** Venn plots of SNAP binding genes in two biological replicates. Rep: replicate. ChIP-seq analysis identified 4372, 4730, 4350, 4949, 3835, 2177, 3020 and 3600 binding genes for SNAP1-rep1, SNAP1-rep2, SNAP2-rep1, SNAP2-rep2, SNAP3-rep1, SNAP3-rep2, SNAP4-rep1 and SNAP4-rep2, respectively.

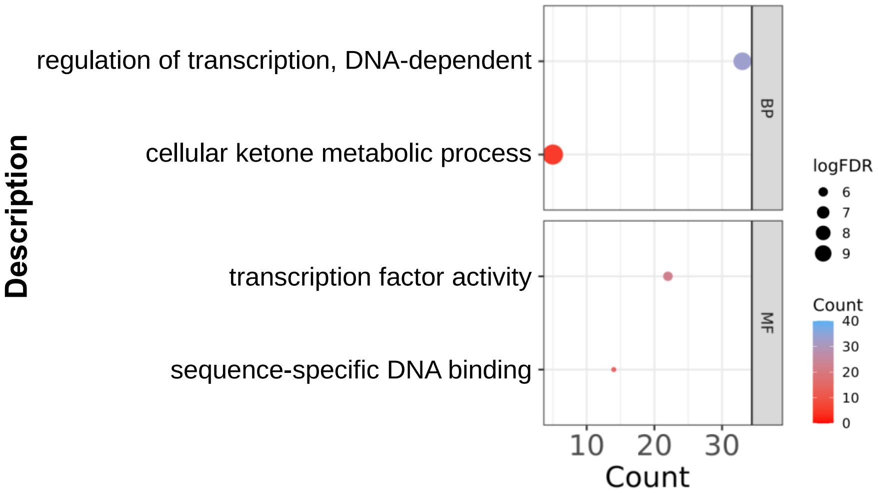


**Supplementary Figure 17.** GO analysis for SNAP1/2/3/4 directly regulated N-responsive genes. Selected GO terms are shown. All significantly enriched GO terms are listed in Supplemental Table 15.

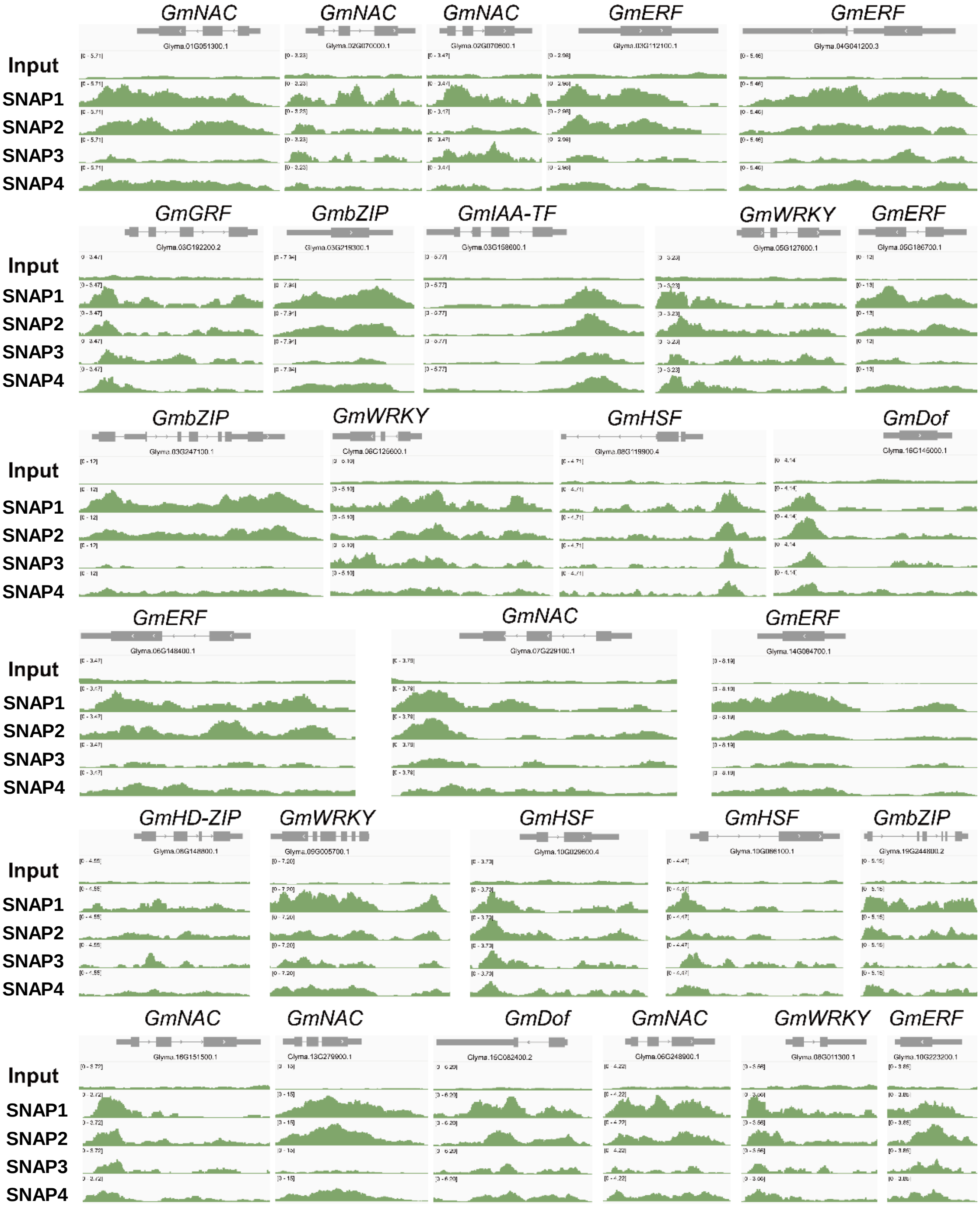


**Supplementary Figure 18.** SNAP1/2/3/4 ChIP peaks and their targeted TFs. NAC: NAM, ATAF, and CUC; ERF: ethylene responsive element binding factor; GRF: growth-regulating factor; bZIP: Basic-leucine zipper; IAA-TF: IAA transcriptional regulator; HSF: heat shock transcription factor; Dof: DNA binding with One Finger; HD-ZIP: homeodomain leucine zipper.

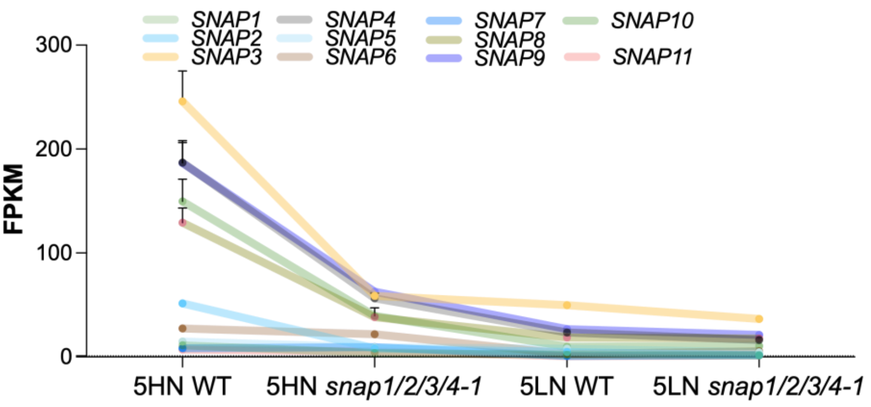


**Supplementary Figure 19.** Normalized expression level of soybean *SNAP* genes in *snap1/2/3/4-1* mutant. Data are represented as mean ± SEM (n= 3 biologically independent experiments). FPKM: Fragments Per Kilobase of exon model per Million mapped fragments. Source data are provided as a Source Data file.

Supplementary Table 1. Primer list.

| Primer name | Forward | Reverse | Target |
|---|---|---|---|
| EF1α-qPCR | TGCAAAGGAGGCTGCTAACT | CAGCATCACCGTTCTTCAAA | qPCR for reference gene GmEF1α (Glyma.17G186600) |
| SNAP1-qPCR | TCCCTCCTCAACAAACCACACTG | AATCTGTGGCATCCAAGAGGTTG | qPCR for SNAP1 (Glyma.02G070000) |
| SNAP2-qPCR | TCCACAAGCCACAATACTCATGTC | AGTCTGTGGCATCCAAGAGATTTG | qPCR for SNAP2 (Glyma.16G151500) |
| SNAP3-qPCR | CCACTATTCAAGGATCAAGAAACTG | GCTAACCTCCAAACAAATCCCAAC | qPCR for SNAP3 (Glyma.16G043200) |
| SNAP4-qPCR | ACCATCTCCAATCGACAACAGC | TGGAGACTGAAGGGTTCAATTGGG | qPCR for SNAP4 (Glyma.19G108800) |
| SNAP1-gene-BP | AAAAAGCAGGCTGCATGGGAACCCCACAATCCAATAACTTGCCACCAGGT | AGAAAGCTGGGTGCTGAAATGGAAGATATTGAGGACTCATGAGTGGCTGCT | BP reaction for SNAP1 (Glyma.02G070000) |
| SNAP2-gene-BP | AAAAAGCAGGCTGCATGGGAACCCCACAATCCAATAACTTGCCACCAGGG | AGAAAGCTGGGTGCTGAAATTGAAGATATTGAGGACTCATGAGTGGCTG | BP reaction for SNAP2 (Glyma.16G151500) |
| SNAP3-gene-BP | AAAAAGCAGGCTGCATGGGAAGCCCAGAATCCAATTTGC | AGAAAGCTGGGTGTCCTTGAAATTGAAGATGAGGACCAAGC | BP reaction for SNAP3 (Glyma.16G043200) |
| SNAP4-gene-BP | AAAAAGCAGGCTGCATGGGAAACCCAGAATCCAATTTGCCACCCGG | AGAAAGCTGGGTGTCCTTGAAATTGAAGATGAGGACCAAGCATTAACCGT | BP reaction for SNAP4 (Glyma.19G108800) |
| SNAP1/2-CRISPR | TGGTCTCgTGCATCTTCACTACCTAAGGAAGAgtttagagctagaaatagc<br>TGGTCTCgAAGTTTGATCCAgttttagagctagaaatagc | TGGTCTCgACTTGTAGATATtgcaccagccgggaatcgaa<br>TGGTCTCgAAACTCCAATACCCTGAAGCTGCTtgcaccagccgggaatcgaa | CRISPR/Cas9 vector construction for SNAP1/2 |
| SNAP3/4-CRISPR | TGGTCTCgTGCAATATCTACAAGTTAGATCCAgtttagagctagaaatagc<br>TGGTCTCgATCATTGCAAGGgttttagagctagaaatagc | TGGTCTCgTGATGTCACTATtgcaccagccgggaatcgaa<br>TGGTCTCgAAACTCTTCACACCAACGCTCTCTtgcaccagccgggaatcgaa | CRISPR/Cas9 vector construction for SNAP3/4 |
| Vector I-sg-1/2/3 | TCTTCACTACCTAAGGAAGA<br>ATATCTACAAGTTTGATCCA<br>AGCAGCTTCAGGGTATTGGA | | three sgRNAs for SNAP1/2 |
| Vector II-sg-1/2/3 | ATATCTACAAGTTAGATCCA<br>ATAGTGACATCATTGCAAGG<br>AGAGAGCGTTGGTGTGAAGA | | three sgRNAs for SNAP3/4 |
| SNAP1-test | CCAATAACTTGCCACCAGGT | AGCGATATTCATGCATGATCCAATTGGTC | Mutant check for SNAP1 (Glyma.02G070000) |
| SNAP2-test | CCAATAACTTGCCACCAGGG | AGTTGAGAATCAGATCCATGATAG | Mutant check for SNAP2 (Glyma.16G151500) |
| SNAP3-test | TTAGGTTCCACCCAACCGAT | ACCTTATTGCTAGTAGGGACATGTG | Mutant check for SNAP3 (Glyma.16G043200) |
| SNAP4-test | AACATCATTTAGCTAGCTAGCC | CGGTTATGCATCACTTGGAAAT | Mutant check for SNAP4 (Glyma.19G108800) |
