# Supplementary material for: The NAC transcription factors SNAP1/2/3/4 are central regulators mediating high nitrogen responses in mature nodules of soybean
Source: Nat Commun. 2023 Aug 5;14:4711. doi: 10.1038/s41467-023-40392-w (PMC10404276; doi:10.1038/s41467-023-40392-w)
Supplement: Supplementary file 3 — Description of Additional Supplementary Files [file 41467_2023_40392_MOESM3_ESM.pdf]

Description of Additional Supplementary Files

Supplementary Data 1: Total expressed genes (FPKM) in mature nodules in time-series HN and LN treatment.

Supplementary Data 2: Co-expression modules for all DEGs in mature nodules in time-series HN and LN treatment.

Supplementary Data 3: GO enrichment analysis of genes in module turquoise.

Supplementary Data 4: GO enrichment analysis of genes in module blue.

Supplementary Data 5: TFs in module blue with GS and total Kin.

Supplementary Data 6: Total DEGs (FPKM) between 5HN and 5LN in mature nodules of W82.

Supplementary Data 7: Total DEGs (FPKM) between 5HN and 5LN in mature nodules of *snap1/2/3/4-1* mutant.

Supplementary Data 8: Total DEGs (FPKM) in mature nodules between W82 and *snap1/2/3/4-1* mutant in 5HN treatment.

Supplementary Data 9: Total DEGs (FPKM) in mature nodules between W82 and *snap1/2/3/4-1* mutant under 5LN treatment.

Supplementary Data 10: GO enrichment analysis of up-regulated genes between W82 and *snap1/2/3/4-1* mutant in 5HN treatment.

Supplementary Data 11: Summary of SNAP TFs binding targets detected by ChIP-seq.

Supplementary Data 12: Predicted motifs in 200bp flanking region of binding summits of SNAP TFs detected by Homer.

Supplementary Data 13: Summary of SNAP directly activated N-responsive genes.

Supplementary Data 14: GO enrichment analysis of SNAP directly regulated genes.
