# Supplementary material for: The NAC transcription factors SNAP1/2/3/4 are central regulators mediating high nitrogen responses in mature nodules of soybean
Source: Nat Commun. 2023 Aug 5;14:4711. doi: 10.1038/s41467-023-40392-w (PMC10404276; doi:10.1038/s41467-023-40392-w)
Supplement: Supplementary file 5 — Reporting Summary [file 41467_2023_40392_MOESM5_ESM.pdf]

nature portfolio

Corresponding author(s): Yuefeng Guan

Last updated by author(s): Jul 4, 2023

Reporting Summary

Nature Portfolio wishes to improve the reproducibility of the work that we publish. This form provides structure for consistency and transparency in reporting. For further information on Nature Portfolio policies, see our Editorial Policies and the Editorial Policy Checklist.

Statistics

For all statistical analyses, confirm that the following items are present in the figure legend, table legend, main text, or Methods section.

n/a	Confirmed	
☐	☒	The exact sample size (n) for each experimental group/condition, given as a discrete number and unit of measurement
☐	☒	A statement on whether measurements were taken from distinct samples or whether the same sample was measured repeatedly
☐	☒	The statistical test(s) used AND whether they are one- or two-sided<br>*Only common tests should be described solely by name; describe more complex techniques in the Methods section.*
☒	☐	A description of all covariates tested
☐	☒	A description of any assumptions or corrections, such as tests of normality and adjustment for multiple comparisons
☐	☒	A full description of the statistical parameters including central tendency (e.g. means) or other basic estimates (e.g. regression coefficient) AND variation (e.g. standard deviation) or associated estimates of uncertainty (e.g. confidence intervals)
☐	☒	For null hypothesis testing, the test statistic (e.g. F, t, r) with confidence intervals, effect sizes, degrees of freedom and P value noted<br>*Give P values as exact values whenever suitable.*
☒	☐	For Bayesian analysis, information on the choice of priors and Markov chain Monte Carlo settings
☒	☐	For hierarchical and complex designs, identification of the appropriate level for tests and full reporting of outcomes
☒	☐	Estimates of effect sizes (e.g. Cohen's d, Pearson's r), indicating how they were calculated

Our web collection on statistics for biologists contains articles on many of the points above.

Software and code

Policy information about availability of computer code

Data collection	The sequence data were generated with Illumina NovaSeq 6000 and Illumina HiSeq X. No software for data collection was used.
Data analysis	RNAseq reads were trimmed with Trimmomatic v0.39 and aligned by HISAT2 v2.1.0; The number of reads mapping to each gene and normalized expression value (FPKM) was calculated by StringTie v1.3.6; Read count was used to perform differentially expression analysis using DESeq2; co-expression network was constructed by WGCNA R package v1.70.3; ChIPseq data were trimmed by trim_galore v0.6.6 and aligned using Bowtie2 v2.3.4.3; Mapped reads were filtered by samtools v1.9; Read coverage was calculated using bamCoverage function in deeptools v3.5.1; Peak calling was performed by MACS2 v2.2.7.1. HOMER v4.11.1 was applied to predict the enriched motifs for candidate peaks with default parameters. Peak annotation was conducted by annotatePeaks.pl script in Homer.

For manuscripts utilizing custom algorithms or software that are central to the research but not yet described in published literature, software must be made available to editors and reviewers. We strongly encourage code deposition in a community repository (e.g. GitHub). See the Nature Portfolio guidelines for submitting code & software for further information.

Data

Policy information about availability of data

All manuscripts must include a data availability statement. This statement should provide the following information, where applicable:

- Accession codes, unique identifiers, or web links for publicly available datasets
- A description of any restrictions on data availability
- For clinical datasets or third party data, please ensure that the statement adheres to our policy

The RNAseq data and ChIPseq data used in this study have been deposited into NCBI database under accession number PRJNA674706 and PRJNA907509. Processed data have been deposited in the NCBI GEO database under the accession number GSE236557.

Human research participants

Policy information about studies involving human research participants and Sex and Gender in Research.

Reporting on sex and gender	N/A
Population characteristics	N/A
Recruitment	N/A
Ethics oversight	N/A

Note that full information on the approval of the study protocol must also be provided in the manuscript.

Field-specific reporting

Please select the one below that is the best fit for your research. If you are not sure, read the appropriate sections before making your selection.

☒ Life sciences ☐ Behavioural & social sciences ☐ Ecological, evolutionary & environmental sciences

For a reference copy of the document with all sections, see nature.com/documents/nr-reporting-summary-flat.pdf

Life sciences study design

All studies must disclose on these points even when the disclosure is negative.

Sample size	For phenotype collection, at least seven individual plants or nodules were taken. No statistical method was used to predetermine sample size. Because it is not a population study and the sample size was sufficient for comparison according to the reports in related research works.
Data exclusions	No data was excluded in this work.
Replication	For ChIPseq, two biological replicates were performed. For RNAseq, three biological replicates were performed. All replications were reliably reproduced and described in figure legend and method
Randomization	The samples used in this study were randomly sampled.
Blinding	No blinding was used. Blinding was not appropriate as the author who performed the experiment and analyzed the data in the meantime.

Reporting for specific materials, systems and methods

We require information from authors about some types of materials, experimental systems and methods used in many studies. Here, indicate whether each material, system or method listed is relevant to your study. If you are not sure if a list item applies to your research, read the appropriate section before selecting a response.

Materials & experimental systems

n/a	Involved in the study
☐	☒ Antibodies
☒	☐ Eukaryotic cell lines
☒	☐ Palaeontology and archaeology
☒	☐ Animals and other organisms
☒	☐ Clinical data
☒	☐ Dual use research of concern

Methods

n/a	Involved in the study
☐	☒ ChIP-seq
☒	☐ Flow cytometry
☒	☐ MRI-based neuroimaging

Antibodies

Antibodies used	FLAG antibody (Sigma, F1804, 1mg/ml)
Validation	FLAG antibody (Sigma, F1804) is commercial and has been validated to work in soybean (https://doi.org/10.3389/fpls.2021.629069)

ChIP-seq

Data deposition

☒ Confirm that both raw and final processed data have been deposited in a public database such as GEO.

☒ Confirm that you have deposited or provided access to graph files (e.g. BED files) for the called peaks.

Data access links<br>*May remain private before publication.*	https://www.ncbi.nlm.nih.gov/bioproject/PRJNA907509<br>https://www.ncbi.nlm.nih.gov/geo/query/acc.cgi?acc=GSE236557
Files in database submission	Files were provided in supplementary Data files.
Genome browser session<br>(e.g. UCSC)	No longer applicable.

Methodology

Replicates	Two replicates of transgenic hairy root tissues with nodules were used.
Sequencing depth	At least 17 million pair-end reads with read length of 150 were generated for each sample.
Antibodies	FLAG antibody (Sigma, F1804).
Peak calling parameters	Peak calling was performed by MACS2 60 v2.2.7.1 with parameters of -q 0.05 and -g 1.0e9.
Data quality	We followed the standard pipeline to analyze the data. Peaks detected in two biological replicates were used for downstream analysis. Detailed information were described in methods.
Software	ChIPseq data were trimmed by trim_galore v0.6.6 and aligned using Bowtie2 v2.3.4.3; Mapped reads were filtered by samtools v1.9; Read coverage was calculated using bamCoverage function in deeptools v3.5.1; Peak calling was performed by MACS2 v2.2.7.1.
